# Approximating defense mechanisms in a national study of adults: prevalence and correlates with functioning

**DOI:** 10.1038/s41398-022-02303-3

**Published:** 2023-01-23

**Authors:** Carlos Blanco, Leonie Kampe, Melanie M. Wall, Shang-Min Liu, Shuai Wang, Eve Caligor, Mark Olfson

**Affiliations:** 1 National Institute on Drug Abuse, Bethesda, MD 20892 USA; 2 Psychologische Hochschule Berlin, Am Köllnischen Park 2, 10179 Berlin, Germany; 3 KlinikumItzehoe, Robert-Koch-Straße 2, 25524 Itzehoe, Germany; 4 Department of Psychiatry, Columbia University/New York State Psychiatric Institute, 1051 Riverside Drive, Unit 69, New York, NY 10032 USA

**Keywords:** Psychiatric disorders, Human behaviour

## Abstract

Despite the clinical relevance of defense mechanisms, there are no published studies in nationally representative samples of their prevalence, correlates, and association with psychosocial functioning. We sought to estimate the prevalence and correlates of 12 defense mechanisms in the general adult population by approximating from items used to assess personality traits in the National Epidemiological Survey on Alcohol and Related Conditions (NESARC), a representative sample of US adults (*N* = 36,653). We examined the associations between sociodemographic characteristics and prevalence of 3 types of defenses mechanisms (pathological, immature, and neurotic). For each defense mechanism, we used the Short-Form 12 to compare psychosocial functioning among 3 groups: those who (1) endorsed the mechanism with self-recognized impairment, (2) endorsed the mechanism without self-recognized impairment, and (3) did not endorse the defense mechanism. The prevalence of defense mechanisms ranged from 13.2% (splitting) to 44.5% (obsessive/controlling behavior). Pathological defenses were more strongly associated with immature defenses (OR = 5.4, 95% CI = 5.2–5.6) than with neurotic defenses (OR = 2.0, 95% CI = 1.9–2.0), whereas the association between immature and neurotic defenses had an intermediate value between the other two (OR = 2.2, 95% CI = 2.1–2.2). Pathological and immature defenses were associated with younger age, having been never married, lower educational attainment, and lower income. After adjusting the crude results for age and sex, individuals who did not endorse a given defense generally had higher scores on the mental health component of the SF-12 than those who endorsed the defense without self-recognized impairment who, in turn, had on average higher scores than those with self-recognized impairment. These results suggest that neurotic, immature, and pathological defense mechanisms are prevalent in the general population and associated with psychosocial impairment. Recognizing defense mechanisms may be important in clinical practice regardless of treatment modality.

## Introduction

Defense mechanisms, defined as mostly unconscious mental operations as an individual’s automatic psychological responses to internal or external stressors or emotional conflict [1]. They serve to keep unpleasant affects, conflicts, or mental states out of awareness and help coping with emotional distress. However, it has been shown that there are differences among the defense mechanisms in terms of their adaptiveness. The concept of defense mechanisms (also known as defenses) is central in psychoanalysis, psychodynamic psychiatry, and psychology [2]. It spans theory, clinical practice, and research, and has even been incorporated into everyday discourse. Empirical research to-date has largely relied on clinical patient studies and convenience samples, reporting the prevalence of individual defenses, defense levels, categories, and styles, as well as their association with symptom and functioning variables [2–4]. Treatment studies have also demonstrated the change characteristics of defenses in relationship to clinical outcome variables. Despite their clinical importance and privileged position in psychodynamic models of psychopathology [5] and development, to our knowledge, no published study has investigated defense mechanisms in a large, general population sample.

A better understanding of defense mechanisms could help advance theory, guide clinical care and improve our understanding of their function. For example, knowing whether immature or pathological defense mechanisms are prevalent in the general population or appear restricted to a small number of individuals would inform whether they are common psychological phenomena or markers of severe psychopathology. Similarly, knowing if they are associated with changes in psychological functioning could indicate whether they should be targets of therapeutic interventions. Furthermore, examining whether there are differences in the relationship between individual defense mechanisms and level of psychosocial functioning may support the existence of a hierarchy of adaptiveness, as postulated by psychoanalytic theory and studies of clinical and non-clinical samples.

An extensive review of the literature on defense mechanisms identified empirical support for the following characteristics: defenses function mostly outside of awareness, develop in a predictable order as individuals mature, are present in the healthy personality, become increasingly used in times of stress, reduce the conscious experience of negative emotions, and, when used excessively, are associated with psychopathology [6]. Several studies have shown that defense mechanisms can be arranged hierarchically based on their association with the level of functioning of the individual [4, 7], concluding that, with the exception of mature defenses, their use is generally associated with lower psychosocial functioning [2, 8, 9].

A landmark prospective cohort study of 268 Harvard sophomores observed their defensive operations over the course of a 25-year period [4, 10]. Based on the results, a hierarchy of adaptiveness of defenses was developed [4] ranging from adaptive mechanisms over neurotic mechanisms to most rigid and most rigid and maladaptive mechanism. The entire spectrum of individual defenses was assessed among healthy subjects and therefore were not necessarily pathological or exclusively found in adults with psychopathology. At the same time, adaptiveness of the individual’s predominant pattern of defense mechanisms defensive style predicted overall psychosocial adjustment [10]. Building on this work, a second study evaluated defense mechanisms in 306 inner-city men at age 47, followed as part of a 50-year longitudinal study, to validate their hierarchy of defenses [7]. Based on this and additional prior work [11], it has been proposed that defenses can be organized hierarchically in four levels [4]; the least functional are the *pathological* defenses, which involve gross distortion of reality [12, 13] and are strongly related conceptually to severe psychopathology; next are *immature* defenses, which distort interpersonal reality and have been found to be most prominent in personality disorders [14]; *neurotic* defenses are intrapsychic mechanisms that relate to psychological suffering when used inflexibly and with rigidity; and, finally most adaptive, *matur*e defenses are strategies for coping and can be used flexibly and sometimes consciously. Other studies have compared the use of defense mechanisms in patients experiencing external stressors to community comparison groups. For example, Perry et al. compared defensive functioning in a sample of mothers with a recent history of breast cancer to a matched sample of healthy mothers in their local community [15].

A number of basic questions about defense mechanisms remain. First, most studies on defense mechanisms have relied on clinical or other samples for which the degree of generalization to the whole adult population is unknown [1–3, 16–18]. Second, the use of clinical samples does not permit estimation of the prevalence and correlates of defense mechanisms in the general population. Third, while clinical work suggests that certain individuals recognize that their use of defense mechanisms is associated with impairment in their daily life, others do not or may not be aware of their uses of defenses. We are not aware of any studies that have examined these questions in a nationally representative sample. Based on previous studies, we hypothesized that: (1) the prevalence of pathological, immature, and neurotic mechanisms of defense would be each 25% or greater; and (2) use of immature and pathological defense mechanisms would be associated with lower psychosocial functioning.

Although defense mechanisms typically operate outside of awareness, their impact on an individual’s behavior, thoughts, and feelings can be observed and assessed, and used to approximate underlying defense mechanisms [2–4, 10, 19]. In the following analyses, we used items collected as part of the National Epidemiological Survey on Alcohol and Related Conditions (NESARC) a large, nationally representative sample, to approximate mechanisms of defense, estimate their prevalence and correlates in the general population, examine whether defense mechanisms are associated with lower psychosocial functioning, and test whether those who recognize impairment have lower psychosocial impairment than those who did not. Although the NESARC was not designed to examine defenses and did not include any direct validation of those constructs (in contrast with studies devised to examined defense mechanisms) [2, 11, 19], we sought to examine whether our approximations had convergent validity by examining their correlation with other measures of functioning.

## Methods

### Sample

The 2001–2002 NESARC (Wave 1) and the 2004–2005 follow-up (Wave 2) is a nationally representative sample of the noninstitutionalized adult US population conducted by the US Census Bureau, under the direction of the National Institute on Alcoholism and Alcohol Abuse, as described elsewhere [20]. The Wave 1 response rate was 81%. Excluding ineligible respondents (e.g., deceased), the Wave 2 response rate was 86.7%, resulting in a cumulative response rate of 70.2% (*n* = 34,653). Wave 2 NESARC weights include a component that adjusts for non-response, demographic factors, and psychiatric diagnoses, to ensure that the Wave 2 sample approximated the target population, that is, the original sample minus attrition between the two waves [20].

### Assessment

All NESARC participants were assessed with the AUDADIS-IV, the NIAAA Alcohol Use Disorder and Associated Disabilities Interview Schedule-DSM IV Version (AUDADIS-IV), a valid and reliable fully structured diagnostic interview designed for use by professional interviewers. Fully structured interviews, rather than clinical assessments, are generally used in large epidemiological studies to ensure their feasibility and decrease procedural variation across interviewers and associated potential biases. A senior psychotherapy researcher and clinician (LK) reviewed the AUDADIS-IV to identify items that could be used to assess underlying defensive operations. Candidate items were then subject to a second-level review and confirmation by a training and supervising analyst (EC) with extensive experience in psychotherapy research and psychoanalytic practice. This process resulted in extraction of items that could be used to approximate 12 defense mechanisms (psychotic distortion, delusional projection, autistic fantasy, projection, withdrawal, acting out, splitting, idealization, devaluation, omnipotence, isolation of affect, obsessive/controlling behavior, and intellectualization (see Appendix in the Supplementary Material for items). All identified items were found in the personality disorders section of the interview and were asked in binary form (yes/no). If an item was endorsed, respondents were queried as to whether the item caused impairment to them. Defense mechanisms were rated as present if one or more of their constituent items were endorsed. Individuals endorsing items assessing defense mechanisms were asked whether the items representing defense mechanisms had interfered with their relations with family or friends, or at work. Thus, for each defense mechanism, individuals were classified into three levels: no endorsement of the defense mechanism, endorsement without self-recognized impairment, and endorsement with self-recognized impairment.

Based on the classification system developed by Vaillant [4, 10], and furthermore informed by the DSM-IV Defensive Functioning Scale [3], these items were subordinated into three levels of adaptiveness (pathological, immature, and neurotic). The pathological category accounted for 2 of the 12 defense mechanisms, the immature category accounted for 8, and the neurotic category for the remaining 2. As expected, since items were extracted from the section of personality disorders, no mature defense mechanisms were assessed in the survey.

Psychosocial functioning was assessed using the Short-Form 12 version 2 (SF-12) [21], a 12-item measure that is a reliable and valid measure of disability used in population surveys [22–24]. The SF-12 is normed to have a mean = 50 and SD = 10. Higher scores indicate better psychosocial functioning. In line with previous reports, we focused on the Mental Component Summary (MCS) because of its particular relevance for overall mental health and functioning, rather than focusing on specific diagnoses. The MCS applies regression weights to the all of the SF-12 items to derive a synthetic measure of mental health [22]. In addition, all NESARC respondents were asked to report their race-ethnicity, age, marital status, educational achievement, and individual income.

### Statistical analyses

Weighted prevalence of mechanisms of defense were estimated for the overall sample and stratified by sociodemographic characteristics. Others Risk differences were used to compare the prevalence of defense mechanisms by sociodemographic group. They were considered to be significant if their 95% did not include 0. T-tests were used to compare scores on the SF-12 between those with and without each defense mechanism. Linear regressions, yielding adjusted means, were used to compare SF-12 scores adjusting for age, sex, and race/ethnicity. No adjustments were made for marital status, educational attainment, or income as those might be influenced by the presence of the defense mechanisms and result in collider bias [25]. Statistical significance was set at 0.05. Furthermore, for all analyses, we considered two point estimates (e.g., prevalence estimates, ORs, scores on the MCS of the SF-12) to be significantly different if their 95% CI did not overlap.

## Results

There was a broad range of variation in the prevalence of the defense mechanisms. When the impairment criterion was not applied, the prevalence ranged from 13.2% (splitting) to 44.5% (obsessive/controlling behavior). Use of at least one neurotic defense was endorsed by 53.6% of respondents, while use of at least one immature defense was endorsed by 49.5%, and at least one pathological defense by 39.4%.

**Fig. 1:**
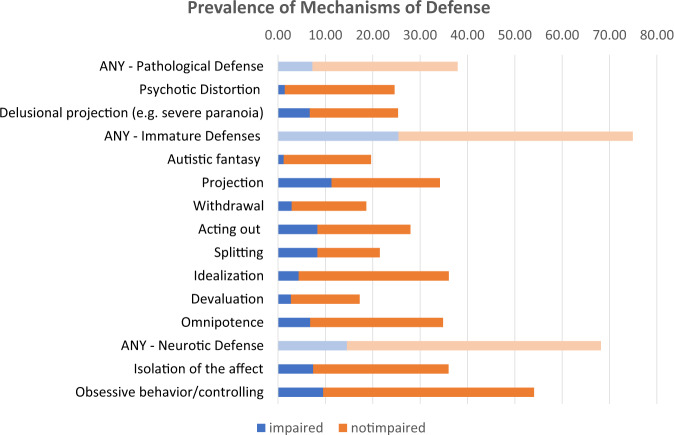
Prevalence of mechanisms of defense in the National Epidemiological Survey on alcohol and related conditions.

Applying the impairment criterion resulted in lower prevalence for all mechanisms of defense, but still considerable variability in prevalence across mechanisms from 1.2% (autistic fantasy) to 11.3% (projection). Use of at least one immature defense (25.4%) was more common than use of at least one neurotic defense (14.6%), which in turn remained more common than use of at least one pathological defense (7.3%). Figure 1 depicts the prevalence of mechanisms of defense when the impairment criterion was not applied (full bar), as well as the prevalence when the impairment criterion was applied (blue portion of the bar). The prevalence of mechanisms of defense that did not meet the impairment criterion is represented by the orange portion of the bar.

The OR of the association between pathological and immature defenses 5.4 (95% CI = 5.2–5.6), whereas the OR of the association of pathological and neurotic defenses was 2.0 (95% CI = 1.9–2.0), and the OR of association between immature and neurotic defenses was 2.2 (95% CI = 2.1–2.2).

Pathological defenses were associated with younger age, having been never married, lower educational attainment, and lower income. Immature defenses were associated with younger age, having been never married, and income between $10,000 and $24,999 (Table 1).

**Table 1:** Association of demographic characteristics with three classes of mechanism of defense in the National Epidemiologic Survey on Alcohol and Related Conditions (*N* = 34,653).

			Any pathological defense (*n* = 13,968)				Any immature defense (*n* = 26,711)				Any neurotic defense (*n* = 23,432)			
		*N*	Row %	Risk Diff	95% CI		Row %	Risk Diff	95% CI		Row %	Risk Diff	95% CI	
Age, years	18–30	5471	45.1	11.2	10.2	12.2	87.1	19.3	18.3	20.2	69.0	0.7	−0.2	1.7
	31–40	6866	39.4	5.4	4.7	6.1	78.8	11.0	10.1	11.9	67.0	−1.2	−2.1	−0.3
	41–50	7424	38.3	4.3	3.6	5.1	75.0	7.2	6.4	8.1	68.8	0.6	−0.3	1.4
	51+	14892	34.0	Ref			67.8	Ref			68.2	Ref		
Gender	Female	20089	39.4	3.1	2.4	3.7	71.9	−6.4	−7.1	−5.8	68.1	−0.2	−0.9	0.5
	Male	14564	36.4	Ref			78.3	Ref			68.4	Ref		
Race	Black	6587	52.7	17.7	16.7	18.6	87.0	14.8	14.1	15.5	67.1	−2.8	−3.8	−1.9
	Native American	578	46.5	11.5	8.4	14.6	81.2	9.0	6.3	11.7	69.0	−1.0	−3.9	2.0
	Asian	968	32.2	−2.8	−3.6	−1.9	71.4	−0.7	−1.9	0.5	61.4	−8.6	−9.7	−7.4
	Hispanic	6359	42.7	7.7	6.7	8.6	80.7	8.5	7.8	9.3	61.2	−8.7	−9.5	−7.9
	White	20161	35.0	Ref			72.2	Ref			69.9	Ref		
Marital status	Never married	6638	45.8	11.2	10.3	12.1	88.0	17.3	16.6	17.9	68.8	0.6	−0.3	1.4
	Divorced/separated/widow	9149	42.0	7.3	6.5	8.2	77.3	6.6	5.9	7.4	67.6	−0.6	−1.4	0.1
	Married	18866	34.6	Ref			70.7	Ref			68.3	Ref		
Education	High school or less	13699	38.8	1.4	0.7	2.1	75.4	0.7	−0.1	1.4	64.2	−6.5	−7.3	−5.8
	Some college or more	20954	37.4	Ref			74.7	Ref			70.7	Ref		
Income, $	0–9999	7924	42.9	12.1	11.2	13.0	74.6	2.3	1.4	3.2	67.2	−3.9	−4.9	−3.0
	10,000–24,999	10496	41.2	10.4	9.6	11.3	76.7	4.4	3.5	5.3	66.6	−4.5	−5.3	−3.6
	25,000–49,999	9745	36.0	5.2	4.4	6.1	75.3	3.0	2.2	3.8	68.7	−2.4	−3.2	−1.5
	≥50,000	6488	30.7	Ref			72.3	Ref			71.1	Ref		

In the overall sample, the mental health component summary had a mean = 51.4 and a standard deviation = 9.5. After adjusting the crude results for age and sex, individuals who did not endorse a given defense had on average higher scores than those who endorsed the defense without impairment who, in turn, had on average higher scores than those with impairment. The only exception to this pattern was for obsessive/controlling behavior, in which individuals who did not endorse this defense had higher scores than those who endorsed it with impairment, but lower scores than those who endorse it without impairment (Table 2).

**Table 2:** Mental Component Summary Score of the Short-Form 12 (SF-12) in individuals with and without mechanisms of defense in the National Epidemiologic Survey on Alcohol and Related Conditions (*N* = 34,653).

	*N*	Mental Component Summary Score									No mechanism of defense versus mechanism without impairment		Mechanism of defense with versus without impairment	
		No mechanism of defense			Mechanism of defense without impairment			Mechanism of defense with impairment			Adjusted beta^a^	*p*-value	Adjusted beta^a^	*p*-value
Mechanism of Defense		Mean	95% CI		Mean	95% CI		Mean	95% CI					
Any Pathological	13,968	52.8	52.7	52.8	50.7	50.6	50.8	43.2	42.9	43.5	2.0	<0.0001	9.5	<0.0001
Psychotic distortion	8961	52.0	51.9	52.0	50.3	50.1	50.4	42.5	41.7	43.3	1.6	<0.0001	9.3	<0.0001
Delusional projection	9637	52.6	52.6	52.7	49.7	49.5	49.8	42.9	42.5	43.3	3.0	<0.0001	9.6	<0.0001
Any Immature	26,711	53.4	53.2	53.5	52.4	52.4	52.5	47.6	47.4	47.7	1.1	<0.0001	5.8	<0.0001
Autistic fantasy	7615	51.8	51.7	51.9	50.4	50.2	50.5	43.9	43.3	44.6	1.3	<0.0001	7.8	<0.0001
Projection	13,073	52.4	52.3	52.5	50.9	50.8	51.0	46.9	46.7	47.1	1.5	<0.0001	5.2	<0.0001
Withdrawal	6936	52.1	52.0	52.1	49.8	49.6	49.9	43.8	43.3	44.2	2.3	<0.0001	8.2	<0.0001
Acting out	9996	52.2	52.1	52.2	51.1	50.9	51.2	45.8	45.6	46.1	1.4	<0.0001	6.5	<0.0001
Splitting	8089	52.5	52.4	52.6	49.3	49.1	49.5	44.8	44.5	45.1	3.2	<0.0001	7.6	<0.0001
Idealization	13,184	52.0	51.9	52.1	51.1	51.0	51.2	45.1	44.7	45.4	0.9	<0.0001	6.8	<0.0001
Devaluation	6637	51.9	51.8	51.9	50.1	50.0	50.3	45.6	45.2	46.0	2.0	<0.0001	6.4	<0.0001
Omnipotence	12,650	51.9	51.9	52.0	51.3	51.1	51.4	47.3	47.0	47.5	0.9	<0.0001	4.9	<0.0001
Any Neurotic	23,432	52.2	52.1	52.3	52.0	52.0	52.1	47.5	47.3	47.7	0.2	0.26	4.6	<0.0001
Affect isolation	12,686	52.1	52.0	52.2	51.3	51.2	51.4	46.1	45.8	46.4	1.1	<0.0001	6.2	<0.0001
Obsessive behavior/controlling	18,293	51.7	51.6	51.8	52.0	51.9	52.1	48.0	47.8	48.3	−0.5	0.0006	3.4	<0.0001

^a^Adjusted for age, sex, and race/ethnicity.

Adjusted differences between individuals who did and did not endorse a given defense without self-recognized impairment ranged from obsessive/controlling behavior (−0.5) to splitting (3.2). Differences between individuals who either endorsed a defense with self-recognized impairment or did not endorse it ranged from obsessive/controlling behavior (3.4) to delusional projection (9.6).

## Discussion

In a large, nationally representative sample of US adults, more than 25% used at least one of the defense mechanisms and use of any one defense mechanism increased the likelihood of using others. Although most adults endorsing a defense mechanism did not believe that it interfered with their work or relations with family or friends, there was a gradient in the level of psychosocial functioning from no endorsement of the defense to endorsement without self-recognized impairment to endorsement with self-recognized impairment. These findings provide new insights into the distribution and functioning of defense mechanisms at the population level.

To our knowledge, this is the first study to assess the prevalence of defense mechanisms in a nationally representative sample. We found that defense mechanisms were widespread. While there are no other population-based studies to compare results, our findings are consistent with clinical experience and with studies of clinical samples and smaller non-clinical samples [1, 2, 9, 26]. For example, a study that examined mechanisms of defense in women with breast cancer and a comparison group of women with no breast cancer found that, among the comparison group, all of them used at least one neurotic defense and 96.2% of them at least one immature defense. The pervasive use of defense mechanisms at all levels of adaptive functioning suggests that they constitute essential intrapsychic operations. Because defenses can become more adaptive and functional as individuals mature, and are modifiable through treatment, recognizing and learning how to work with defenses appears to be an essential skill for clinical practice [18, 26].

There was a broad range in the prevalence of individual defenses, consistent with previous findings that individuals use a variety of approaches to manage the relationship between their internal world and external reality [27]. The use of specific defenses and their level of adaptiveness probably results from a combination of life events, cultural environment, genetic predisposition, and the ability of individuals to modify these factors through maturation, insight, and the nurturing of others [26, 28]. For example, as compared to a community comparison group, women with breast cancer were more likely to use lower-level defensive styles than the comparison group, suggesting that stressful events may influence the type of defenses used by individuals.

Pathological and immature mechanisms of defense were inversely associated with age, whereas differences in the age distribution of neurotic defenses were less marked. These findings are consistent with smaller, longitudinal studies that have also found an inverse association between age and level of defenses [4, 6, 10]. Because defenses cannot influence age (i.e., reverse causation is not possible), these results suggest that as individuals age they are less likely to use lower-level defenses. An alternative and in our view less likely explanation is that as individuals age, they become less aware of their use of pathological and immature defenses.

Women were more likely than men to endorse pathological and immature defenses, but equally likely to endorse neurotic defenses. Sex differences in defenses could be due to differences in development [29, 30], cultural expectations, or accepted social norms [31], or be the result of adaptations to situations in which women are victims of discrimination of violence [32]. They may also reflect differences in expression, self-awareness, or willingness to acknowledge certain behaviors [33]. Sex differences in the expression of psychopathology have been extensively documented [34–36]. Nevertheless, because we are not aware of any other studies that have examined sex differences in defenses, we believe these findings should be considered tentative and in need of replication.

Use of a given defense increased the probability of using any other defenses, yielding a potentially high number of combinations of defenses and clinical presentations. In these combinations, defenses that were closer to each other on the adaptation hierarchy (e.g., pathological and immature) were more strongly associated than those hypothesized to be more distantly related (e.g., pathological and neurotic). These findings are in accord with a hierarchy of adaptiveness of defensive operations, and with clinical experience and clinical research [36] suggesting that as individuals progress in treatment, defenses tend to shift gradually, moving up to the next higher level of adaptation. The observed patterns are also consistent with the reports that a given defensive style generally, although not always, is substituted by defenses closer in the hierarchy of adaptation than by those more distantly related [26].

As compared to individuals who used a given defense mechanism, those who did not use it had on average higher psychosocial functioning. For each defense, most individuals endorsing it did not consider that the defense interfered with their life, suggesting that even when individuals are aware of using a particular defense, they often remain unaware of the potential maladaptive nature of their behavior. This frequent lack of insight is in line with the psychodynamic view and clinical experience that defense mechanisms are generally ego syntonic, exerting a toll on psychosocial functioning that typically remains outside of the awareness of those using them. It is only as defenses become more extreme, inflexible, and pervasive that the individual is likely to become aware of their associated functional impairment and the defense becomes ego dystonic [26]. One exception to this pattern may be obsessive/controlling behavior. It is possible, for some individuals or when used at relatively low levels, that use of that defense may, may mimic some aspects of mature defenses and help increase psychosocial functioning. However, for others or when used excessively, it may reverse and begin to degrade psychosocial functioning. Future research should investigate under what circumstances or at what levels use of this defense leads to improved functioning.

From the clinical point of view, the widespread use of defense mechanisms suggests that they are likely to manifest themselves not only in psychotherapy, but also during pharmacological or other types of treatment and in interactions with other health care professionals. The clinical reality that patients may be unaware of the extent of their use of maladaptive defenses highlights the need to evaluate defensive functioning in patients who present in distress or having unexpected difficulties coping. Severity of impairment in the setting of maladaptive use of defenses might determine the need for treatment by individuals with expertise in psychodynamic psychotherapy or other approaches directed at improving defensive operations.

From the theoretical point of view, our findings are consistent with the hypothesized central role of defenses in intrapsychic operations and in determining behavior. They are also consistent with predictions that, despite variability in impairment within each level of defenses, lower-level defenses are generally associated with greater psychosocial impairment than higher level ones. Future research should investigate whether these findings hold for mature defenses in the general population and the mechanisms that lead to defense-associated functional impairment. Twin and other family-based designs could shed light on the role of genetic and environmental contributions to the development and use of defense mechanisms.

This study has several limitations. First, defense mechanisms, which are complex constructs, were approximated using items developed to assess personality disorder criteria rather than defenses per se. In some cases, such as the assessment of obsessive behavior, the boundary between personality disorder criteria and conscious derivatives of defenses may have been imperfect. Furthermore, as in most large population psychiatric epidemiological studies, items were assessed by a structured interview, rather than by a clinician. Nevertheless, the finding of a gradient in psychosocial functioning from no endorsement of defense mechanism, to use without impairment, to use with impairment supports the face validity of the assessment. Second, only 123 mechanisms could be assessed, most of which were pathological or immature, and mature mechanisms were not assessed. Because immature defenses were rated with a greater number of items, respondents may have been more likely to endorse them than pathological or neurotic defenses that had fewer items. It is possible that assessment of other mechanisms would have yielded a different pattern of results. Furthermore, because the pathological and neurotic categories had only two items, it was not possible to assess the internal consistency of this category or to use the items as indicators or a latent variable. Third, the NESARC did not assess the frequency of use of each defense mechanism. Fourth, because these data consist of cross-sectional associations, they can suggest but not prove any causal relationship between defenses and psychosocial functioning. Finally, because the NESARC did not assess individuals in jails or prisons or inpatient populations, the results may not generalize to several important institutionalized populations.

In conclusion, defense mechanisms are common in the general adult population and are associated with decreased psychosocial functioning. The high prevalence of defenses suggests the centrality of these mental processes and their importance in models of intrapsychic functioning. Recognizing and managing defenses appear essential skills for practicing clinicians regardless of their theoretical orientation.

## Supplementary information

Supplementary Information
