# Supplementary material for: Approximating defense mechanisms in a national study of adults: prevalence and correlates with functioning
Source: Transl Psychiatry. 2023 Jan 23;13:21. doi: 10.1038/s41398-022-02303-3 (PMC9870881; doi:10.1038/s41398-022-02303-3)
Supplement: Supplementary file 1 — Supplementary Information [file 41398_2022_2303_MOESM1_ESM.docx]

Appendix. Assessment of pathological, immature and neurotic mechanisms of defense in participants of the National Epidemiologic Survey on Alcohol and Related Conditions (N=34,653).

| Mechanism of Defense | Assessment Questions |
| --- | --- |
| **Pathological** |  |
| Psychotic Distortion | 1. Have you ever felt that you could make things happen just by making a wish or thinking about them? 2. Have you believed that you have a “sixth sense” that allows you to know and predict things that others can’t? 3. Have you had the sense that some force is around you, even though you cannot see anyone? 4. Have you often thought that objects or shadows are really people or animals, or that noises are actually people’s voices? |
| Delusional projection | 1. When you are around people, do you often feel that you are being watched or stared at? 2. When you’ve been under a lot of stress, have you gotten suspicious of other people or felt   spaced out?   1. Have you often had the feeling that things that have no special meaning to most people are   really meant to give you a message?   1. When you are around people, have you often had the feeling that you are being watched or   stared at?   1. Have you felt suspicious of people, even if you have known them for a while? |
| **Immature** |  |
| Autistic fantasy | 1. Have you thought a lot about the power, fame, or recognition that will be yours someday? 2. Have you thought a lot about the perfect romance that will be yours someday? |
| Projection | 1. Do you often have to keep an eye out to keep people from using you, hurting you or lying to you? 2. Do you spend a lot of time wondering if you can trust your friends or the people you work with? 3. Do you find that it is best not to let other people know much about you because they will use it against you? 4. Do you often detect hidden threats or insults in things people say or do? 5. Have you OFTEN suspected that your spouse or partner has been unfaithful? 6. Have you felt that others are often envious of you? |
| Withdrawal | 1. Have you avoided jobs or tasks that dealt with a lot of people? 2. Do you avoid getting involved with people unless you are certain they will like you? 3. Have you almost always preferred to do things alone rather than with other people? |
| Acting out | 1. Do you flirt a lot? 2. Do you try to draw attention to yourself by the way you dress or look? 3. Have you ever gotten into sexual relationships quickly or without thinking about the consequences? 4. Have you tried to hurt or kill yourself, or threatened to do so? 5. Have you ever cut, burned, or scratched yourself on purpose? 6. Have you gone to extremes to keep people from leaving you? |
| Splitting | 1. Have your relationships with people you really care about had lots of extreme ups and downs? 2. Have you all of a sudden changed your sense of who you are and where you are headed? 3. Have you been so different with different people or in different situations that you sometimes don’t know who you really are? |
| Idealization | 1. Have people often failed to appreciate your very special talents or accomplishments? 2. Have you felt it was important to spend time with people who are important or influential? |
| Devaluation | 1. Have you often found yourself losing interest in people after they have served their purpose? 2. Have other people’s problems or feelings failed to interest you? 3. Have you found that there are very few people who are worth your time and attention? |
| Omnipotence | 1. Have people told you that you have too high an opinion of yourself? 2. Has it been very important to you that people pay attention to you or admire you in some way? 3. Have you thought that you could ignore certain rules or social conventions when they get in your way? 4. Have you felt that you were the kind of person who deserves special treatment? 5. Have you often expected other people to do what you ask without question because of who you are? |
| **Neurotic** |  |
| Affect Isolation | 1. Do you rarely show much emotion? 2. Do you find that nothing makes you very happy or very sad? 3. Have you rarely shown emotion? 4. Have you had trouble expressing your emotions and feelings? |
| Obsessive behavior/controlling | 1. Are you the kind of person who focuses on details, order and organization or likes to make lists and schedules? 2. Do you have trouble finishing jobs because you spend so much time trying to get things exactly right? 3. Do other people think you have unreasonably high standards and morals about what is right and what is wrong? 4. Is it hard for you to let other people help you if they don’t agree to do things exactly the way you want? |
